# Tobacco use in Nigerian youth: A systematic review

**DOI:** 10.1371/journal.pone.0196362

**Published:** 2018-05-03

**Authors:** Bankole K. Oyewole, Victor J. Animasahun, Helena J. Chapman

**Affiliations:** 1 Faculty of Clinical Sciences, Obafemi Awolowo College of Health Sciences, Olabisi Onabanjo University, Sagamu, Nigeria; 2 Lagos State University Teaching Hospital, Lagos, Nigeria; 3 Department of Environmental and Global Health, University of Florida, Gainesville, Florida, United States of America; Edith Cowan University, AUSTRALIA

## Abstract

**Background:**

Tobacco use is the most important preventable cause of premature death and major risk factor for non-communicable diseases. Due to strict tobacco legislation in the western hemisphere, many African nations like Nigeria have shifted from being a tobacco-producing nation to a tobacco-consuming one. The purpose of this study was to systematically review existing literature on tobacco use among Nigerian adolescents and young people and identify the prevalence, distribution and factors influencing of tobacco smoking. These data are necessary to formulate and adapt control measures aimed at tobacco cessation among young people, and preventing long-term smoking behaviors.

**Methods:**

Three databases (African Journals Online, PsychInfo, PubMed) were searched for peer-reviewed publications, published between January 2000 and March 2017. Additional searches were completed on Google Scholar, and other documents and reports of the Nigerian government and the Global Youth Tobacco Survey were consulted. Using the PRISMA guidelines to evaluate studies, we included studies that reported prevalence of tobacco use in adolescents or youths, aged 10 to 24, and excluded evaluations of tobacco-related medical conditions.

**Results:**

A total of 30 studies with a total population of 26,709 were reviewed. Prevalence rates of tobacco smoking ranged from 0.2% to 32.5%. Among the gender-specific studies, the prevalence of smoking among females ranged between 2.2% to 10% while that of males ranged from 1% to 32.5%. Gender distribution among these studies was mixed (80.0%), males only (13.3%) and females only (6.7%). Smoking prevalence was higher among males than females. The most common risk factors for tobacco use included peer influence, family conditions, psychosocial factors and male gender. Additional risk factors included concomitant substance abuse, media advertisements and increasing age.

**Conclusions:**

Tobacco smoking poses a huge burden to Nigerian youths and various determinants were highlighted in this review. It is imperative that all stakeholders engage in concerted efforts to target both in-school and out-of-school youths in tobacco control strategies.

## Introduction

Tobacco smoking is the single most important cause of preventable and premature death globally. The World Health Organization (WHO) estimates that tobacco kills nearly seven million people annually and 100 million deaths were recorded over the course of the 20th century [1]. More than six million of those deaths are the result of direct tobacco use while close to 900,000 deaths are the result of non-smokers being exposed to second-hand smoke. Unless urgent action is taken, the annual death toll could rise to more than eight million by 2030 [1,2]. Close to 80percent of world's one billion smokers live in low to middle income countries like Nigeria [2].

Non-communicable diseases (NCDs) already account for more than 80 percent of premature deaths in developing countries and the single largest preventable risk factor for NCDs is tobacco smoking [3]. While the global burden of NCDs is estimated to rise by 17 percent in the next decade, it is expected that a sharp increase of 27 percent would be experienced in the Africa region [1]. Nigeria is the most populous nation in Africa with a large population of adolescents and young people, which affects health indices across the region. [3–5].

The transition of tobacco hubs from the West to the African continent is of paramount importance, and Nigeria lies at the forefront of the shift from a tobacco-producing to a tobacco-consuming nation [6]. Between 1990 and 2009, cigarette consumption decreased by about 26 percent in western Europe while there was almost a 60 percent increase in tobacco consumption in Africa and Middle Eastern countries [7]. As such, Africa has become a prime target for tobacco companies [8]. Tobacco regulation has been tightened in North America and Europe in contrast to the African region where some countries are either yet to implement tobacco laws or are more susceptible to being influenced by the tobacco lobby groups that see the continent as a vast area for growth [8,9]. The WHO Framework Convention on Tobacco Control (FCTC), was adopted by the 56th World Health Assembly on May 21, 2003, and implemented on February 27, 2005. In this treaty, WHO recommends a four-pronged strategy for the control of smoking [10]. The first prong advocates a ban on all forms of advertising and an increase in public health information with special attention to youths [10]. The Nigeria National Tobacco Control Act of 2015, was passed to domesticate the WHO FCTC; however, implementation has been poor as most public places are yet to be smoke free, and no funds have been dedicated for tobacco law enforcement [11].

Most smokers begin smoking during their adolescent years, and they grow into the habit making nicotine addiction difficult to curb [12]. As these adolescents become adults, they serve as role models to youths, reinforcing a vicious cycle [12]. The health consequences of tobacco smoking depend on the duration and quantity of the smoking behavior. Starting to smoke early in life increases the risk of NCDs, and adolescent smokers are at greatest risk of future morbidity and mortality [13].

Half of adolescent smokers become regular smoking adults, and a further half of this population is expected to die of tobacco-associated illnesses, further highlighting the great burden smoking in young people poses and the need to end this habit [14]. The crucial role young people play in the perpetuation of smoking is not lost on the tobacco industry. Evidence shows a large percentage of their advertising dollars is now spent to encourage young people to smoke, with more than nine billion dollars directed towards this goal every year [15,16]. Approximately 40 percent of Hollywood movies and movies rated for young people depict scenes of smoking, highlighting the shift from obvious tobacco advertisements to more subliminal and harder to regulate spheres [9].

The purpose of this study was to systematically review existing literature on tobacco use among Nigerian adolescents and young people to identify the prevalence, distribution and factors influencing tobacco smoking. These data are necessary to formulate and adapt control measures aimed at tobacco cessation among young people, and prevention of long-term smoking behaviors.

## Methods

### Search strategy and selection criteria

**Fig 1:**
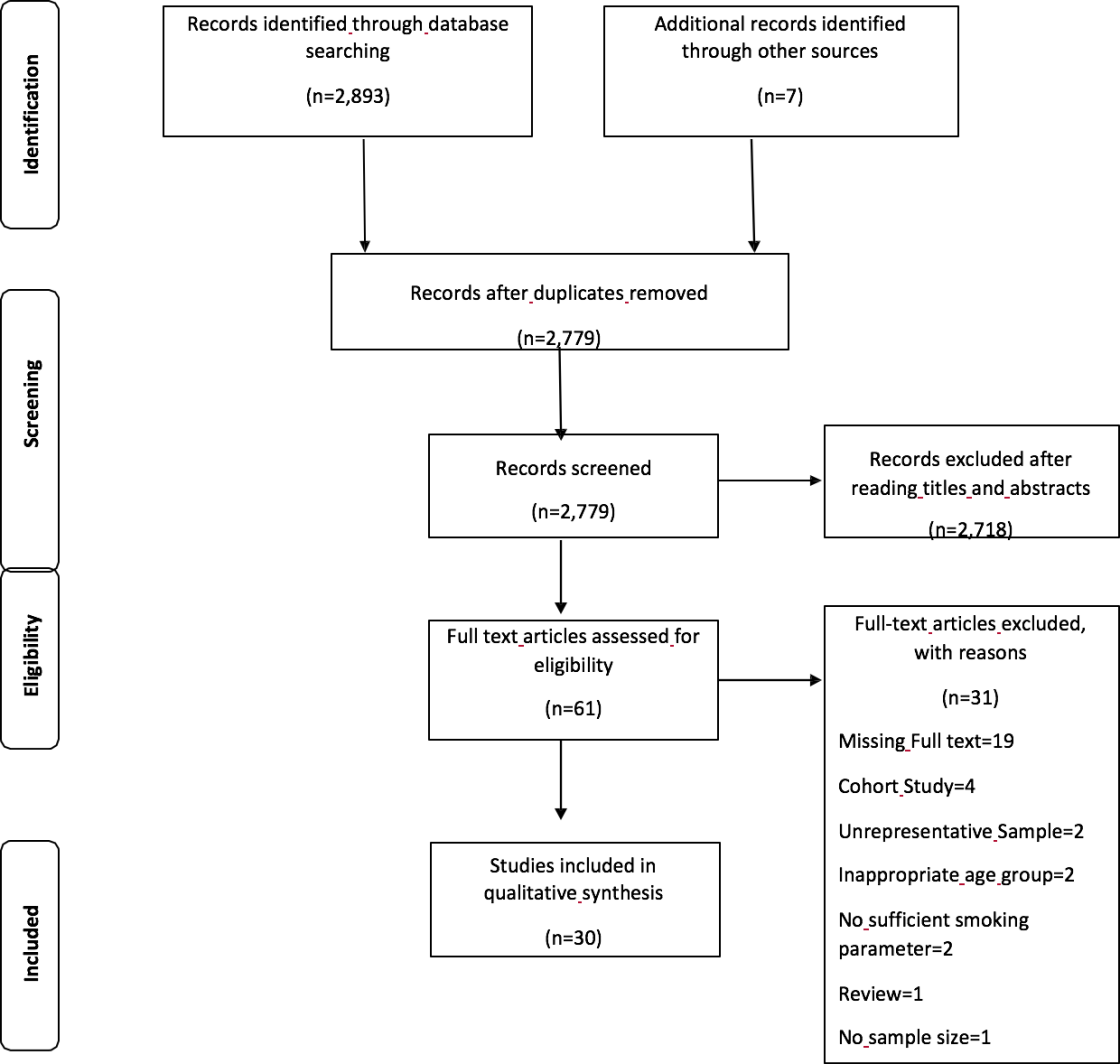
PRISMA flowchart of the search strategy.

A systematic review was conducted, following the Preferred Reporting Items for Systematic Reviews and Meta-Analyses (PRISMA) guidelines for assessments of intervention studies [17]. We aimed to identify studies that examined general tobacco use in Nigeria among “youth”, using the WHO definition of youth as individuals between ages 10 and 24 years [13]. We also consulted the Global Youth Tobacco Survey (GYTS), a school-based survey that examined tobacco use in youth between 13 and 15 years of age, including academic curriculum on tobacco use, exposure to secondhand smoking, media advertisement, tobacco cessation, and availability of and access to tobacco, and guided the assessment of interventions [18]. We defined “current smoker” as youth who had smoked at least once during the previous 30 days [18]. Three researchers searched three databases (African Journals Online, PsychInfo,PubMed), for peer-reviewed publications, published between January 2000 and March 2017. We used key terms, “Nigeria” and “tobacco” or “smoking or “cigarette”, and “adolescent” or “youth” or “school”. References of eligible studies were searched for possible inclusion. We limited our search to tobacco use prevalence in adolescents or youths, aged 10 to 24, excluding the evaluation of tobacco-related medical conditions. We excluded studies that described case reports, reviews, and qualitative or mixed method research designs. We did not include studies published in languages other than English, reported findings from multiple countries, failure to defied smoking behaviors, or conducted outside of Nigeria. Additional searches were completed on Google Scholar and other documents and reports of the Nigerian government and the GYTS were consulted. In initial search, two pairs of researchers (BKO and VJA or BKO and HJC) reviewed all titles and abstracts and excluded studies that did not meet the inclusion criteria. Full-text articles of eligible studies were obtained and evaluated independently by all researchers, based on limits to the inclusion criteria. When full-text articles were unavailable on library databases, we emailed authors and, if necessary, sent reminder emails over a period of six months. Those full-text articles that were unable to be obtained by the respective authors were excluded. Consensus was met by all researchers if there were discrepancies in this evaluation process. Fig 1 presents the PRISMA flowchart of the search strategy. Since this review evaluated the publicly available scientific literature, ethical authorization was not obligatory.

### Data extraction

From the final included articles, authors extracted data in a spreadsheet, including year of data collection period, study location (e.g., site, geopolitical zone, rural or urban setting), sample size, gender (for single gender studies), smoking prevalence, definition of smoking status and determinants or risk factors. A descriptive table was developed to organize the general characteristics of selected studies.

### Quality assessment

Using the following study appraisal criteria, based on a previous systematic review [19], we evaluated the quality of the selected quantitative studies. First, the study population should be representative, including the appropriate selection and size of the sample. Second, the exposure variable, including sampling method and data collection strategy, should be described. Third, the outcome variable should be defined along with the analytical plan to examine associations and level of significance in order to determine tobacco risk factors. These criteria were reviewed by two pairs of researchers (BKO and VJA or BKO and HJC), and consensus was met to include or exclude each study.

## Result

This systematic review focused on studies that investigated the prevalence of tobacco smoking among Nigerian youths between January 2000 and March 2017 (Table 1). Of the total 30 studies, the sample sizes ranged from 128 to 2,408, and the cumulative sample total was 26,709. These studies were conducted in rural (33.3%), urban (56.7%) and mixed settings (10.0%). A total of 3(10.0%) studies were conducted in more than one region and could be said to be nationally representative as respondents were from southern and northern Nigeria, while other studies (90.0%) were city-specific. About three-quarters (76.7%) were conducted in southern Nigeria, and others (13.3%) were conducted in northern Nigeria. The gender distribution among these studies was mixed (80.0%), males only (13.3%) and females only (6.7%). A total of 13 (41.3%) did not include the study date, but were published within the selected time frame for this review.

**Table 1:** General characteristics of selected studies (n = 30).

Author (Publication year)	Study date	Study type	Study location	Geopolitical zone	Sample size	Current smoking prevalence	Gender	Rural or urban setting	Methodology	Determinants or risk factors
Egbuonu et al. (2004)	2001–2002	School-based	Anambra State	South East	725	4.7%	Female only	Rural	Cross-sectional study design; Two-stage, systematic random sampling	None stated
Abdulkarim et al. (2005)	2000	School-based	Kwara State	North Central	1,181	4.8%	Male: 51.9% Female: 48.1%	Urban	Cross-sectional study design; Multi-staged, simple random sampling	Other substance abuse
Omokhodion et al. (2007)	None stated	School-based	Oyo State	South West	1,223	3.4% Male: 5.4% Female: 1%	Male: 53.2% Female: 46.8%	Urban	Cross-sectional study design; GYTS tool	Loneliness: 15% Peer: 14% Stress: 12%
Osungbade et al. (2008)	2003	School-based	Oyo State	South West	416	5.1%	Male: 51% Female: 49%	Rural	Cross-sectional study design; Multi-staged sampling; GYTS tool	Peers (p = 0.00518) Parent (p = 0.002856) Advertisement (p = 0.032989)
Abdulmalik et al. (2009)	None stated	School-based	Borno State	North East	340	19.1%	Male only	Rural	Cross-sectional study design Multi-staged sampling; GSHS tool	Concomitant substance abuse
Aina et al. (2009)	2007	School-based	Lagos State	South West	433	3.93% Male: 7.39% Female: 0.87%	Female: 53.12% Male: 46.88%	Urban	Cross-sectional study design	None stated
Osibogun et al. (2009)	None stated	School-based	Multi-center (Borno, Delta, Ebonyi, Kano, Kogi, Ogun)	North Central North East North West South East South South South West	1,183	17.1% Borno: 16% Delta: 12.6% Ebonyi: 23.7% Kano: 11.3% Kogi: 36.7% Ogun: 2.1%	Male: 71% Female: 29%	Rural and urban	Cross-sectional study design Multi-staged, stratified sampling	Peers: 67.4% TV: 13.4% Low parental education
Adebiyi et al. (2010)	None stated	Out-of-school	Oyo State	South West	215	11.6%	Male: 53% Female: 47%	Rural	Cross-sectional study design; Multi-staged sampling	Male gender Peer pressure Stimulant effect
Ekanem et al. (2010)	2008	School-based	Multi-center (Abuja, Cross River, Ibadan, Kano, Lagos)	North Central South South South West North West	5,459	Abuja: 3.5% Cross River: 4.1% Ibadan: 3.5% Kano: 6.2% Lagos: 2.6%	Abuja (Male: 5.6%; Female: 1.3%) Cross River (Male: 6.8%; Female: 1.2%) Ibadan (Male: 1.4%; Female: 5.5%) Kano (Male: 11.4%; Female: 0.3%) Lagos (Male: 2.8%; Female: 1.8%)	Rural and urban	Cross-sectional study design; Two-stage, cluster sampling; GYTS tool	None stated
Onyiriuka et al. (2010)	2009	School-based	Edo State	South South	1,060	16.5%	Male only	Rural	Cross-sectional study design; Total sampling	Close contact with father Elder sibling or best friend who smokes Increasing age Poor academic performance Peer pressure
Oshodi et al. (2010)	2005	School-based	Lagos State	South West	402	3%	Male: 43.5% Female: 56.5%	Urban	Cross-sectional study design; Multi-staged sampling	Male gender
Adeyeye et al. (2011)	None stated	School-based	Lagos State	South West	1132	12.4%	Males: 51.9% Female: 48.1%	Rural and urban	Cross-sectional study design; Stratified sampling	To imitate friends: 54.2% Curiosity: 15.7% Sign of maturity: 13.6%
Afolabi et al. (2012)	None stated	School-based	Osun State	South West	782	6.3%	Male: 48.5% Female: 51.5%	Rural	Cross-sectional study design; Multi-staged sampling	None stated
Fawibe et al. (2011)	2009	School-based	Kwara State	North Central	1,754	5.7%	Male: 65.5% Female: 34.5%	Urban	Cross-sectional study design; Multi-staged sampling	Male gender Peer pressure Parental smoking Advertisement
Ogunwale et al. (2012)	None stated	Other	Ogun State	South West	128	1%	Male only	Urban	Cross-sectional study design; Total sampling	None stated
Orimadegun et al. (2012)	None stated	School-based	Ogun State	South West	1272	1.6%	Male: 45.8% Female: 54.2%	Rural	Cross-sectional study design	Male gender Increasing age Peer and parental influence Islamic religion
Aigbiremolen et al. (2013)	2012	School-based	Edo State	South South	353	11.6%	Male: 47.9% Female: 52.1%	Urban	Cross-sectional study design; Multi-staged sampling	Poor academic performances
Atoyebi et al. (2013)	2012	School-based	Ekiti State	South West	280	14.6%	None stated	Urban	Cross-sectional study design	None stated
Awopeju et al. (2013)	None stated	School-based	Osun State, Lagos State	South West	675	5.04% Males: 3.4% Females: 1.6%	Male: 48.9% Female: 51.1%	Urban	Cross-sectional study design	None stated
Odukoya et al. (2013)	2009–2010	School-based	Lagos State	South West	973	3.5%	Male: 52.9% Female: 47.1%	Urban	Cross-sectional study design; Multi-staged sampling	Male Being in a private school Poor knowledge of tobacco-related health risks Positive attitude towards smoking Peer and family influence
Odey et al. (2012)	None stated	School-based	Cross River State	South South	375	6.4%	Male: 38.9% Female: 61.1%	Urban	Cross-sectional study design; Multi-stage sampling	None stated
Raji et al. (2013)	2012	School-based	Sokoto State	North West	228	8.3%	Male: 79.4% Female: 20.6%	Rural	Cross-sectional study design; Two-stage sampling; GYTS tool	Smoking father (p = 0.0036) Smoking friend (p = 0.002) Smoking brother (p = 0.002)
Ebirim et al. (2014)	2013	School-based	Imo State	South East	944	11.2%	Male only	Urban	Cross-sectional study design	Peer pressure: 36.1% Family member: 31.9% Curiosity: 31.9%
Oye-Adeniran et al. (2014)	2012	School-based	Oyo State, Kano State	North West South West	2,408	2.2%	Female only	Urban	Cross-sectional study design	None stated
Arute et al. (2015)	2014	School-based	Delta State	South South	400	3.5%	Male: 52% Female: 48%	Urban	Cross-sectional study sample; Multi-stage, random sampling	Relieves stress: 65% Helps me forget my worries: 43% Helps me read better: 14%
Abiola et al. (2016)	None stated	Community	Lagos State	South West	402	14.7%	Male: 63.4% Female: 36.6%	Urban	Cross-sectional study design	Significant associations with initiation of cigarette smoking among teenagers: unmarried, Islam, Yoruba ethnicity, primary school, friends influence, advert influence, parents influence, relatives influence
Adebiyi et al. (2016)	None stated	School-based	Oyo State	South West	544	0.4%	Male: 44.7% Female: 55.3%	Rural	Cross-sectional study design	None stated
Anyanwu et al. (2016)	2015	School-based	Ebonyi State	South East	620	14.4%	Male: 47.9% Female: 52.1%	Urban	Cross-sectional study design; Multi-stage sampling; WHO drug use questionnaire tool	Divorced/separated family structure (p = 0.002) Orphan (p = 0.027)
Idowu et al. (2016)	None stated	School-based	Osun State	South West	476	0.2%	Male: 49.8% Female: 50.2%	Rural	Cross-sectional study design	None stated
Odukoya et al. (2016)	None stated	Other	Lagos State	South West	326	32.5% (Male: 36.6%; Female: 10%)	Male: 84.7% Female: 15.3%	Urban	Cross-sectional study design	Factors associated with current smoking: male gender, lives with peers or lives alone; drinks alcohol; uses marijuana; ever used other forms of tobacco

Abbreviations: WHO = World Health Organization; GYTS = Global Youth Tobacco Survey; GSHS = Global School-based Student Health Survey

Notes: a) The Global Youth Tobacco Survey (GYTS) is a standardized tool of 56 questions, developed by the World Health Organization (WHO) and the Centers for Disease Control and Prevention (CDC), used to monitor tobacco use among youths [World Health Organization. Global youth tobacco survey (GYTS). Geneva, Switzerland: World Health Organization; 2018. http://www.who.int/tobacco/surveillance/gyts/en/]. b) The Global School-based Student Health Survey (GSHS) is a standardized tool, developed by the WHO, used to study health behaviors among youths [World Health Organization. Global school-based student health survey (GSHS). Geneva, Switzerland: World Health Organization; 2018. http://www.who.int/chp/gshs/en/].

Of the total 30 studies, prevalence rates of tobacco smoking ranged between 0.2% and 32.5%. Studies showed that smoking prevalence was higher among males than females [20–27]. Among the gender-specific studies, the prevalence of smoking among females ranged between 2.2% [28] to 10% [27] while that of males ranged from 1% [29] to 32.5% [27].

Although Atoyebi et al. (2013) did not outline risk factors in their study, all tobacco users in that study were males [30]. In particular, two studies had striking prevalence rates of 19.1% in males [31] and 32.5% in males [27]. Three studies reported the prevalence among females as 2.2% [28], 4.7% [32] and 10% [27]. Over one-third (36.7%) of the studies reviewed did not measure risk factors for tobacco smoking in young people. The most common risk factors included peer influence; male gender; family conditions, such as low parental education, polygamy, not living with parents, having a parent who smokes and having divorced or separated parents; and psychosocial factors such as belonging to a polygamous home, low level of father’s education, feeling of loneliness and depressive symptoms, were found to contribute significantly to the prevalence of smoking among these street children. Additional risk factors were concomitant substance abuse, media advertisements and increasing age.

## Discussion

To the best of our knowledge, this is the first systematic review to investigate the prevalence, distribution and influences of tobacco smoking among Nigerian adolescents and young people. This is set against the backdrop of a nation experiencing significant demographic changes with increased populations of young people [33]. The studies were predominantly from southern Nigeria, reflecting the varying research productivity across geopolitical zones [34]. The majority of the studies targeted males only, which is consistent with previous studies [35–37]. The gender difference may be attributed to societal perception as most African communities see smoking as a sign of masculinity or even specific to manhood and vigor [38], while social values discourages smoking among women [35]. Some studies reported prevalence values above the latest WHO national prevalence of tobacco smoking among males (17.4%) and females (1.1%) [1,27,31, 32]. Most studies included in our review, that used the GYTS protocol, targeted the in-school youth, demonstrating a lower prevalence of tobacco use when compared to out-of-school (street).

Abdulmalik et al. (2009) conducted a study in Borno State, North East, Nigeria, among the “Almajiris”, youth between 11 and 16 years of age, who lacked formal education, spent minimal time in Arabic classes, and spent time involved in street begging [31]. Several psychosocial factors, such as belonging to a polygamous home, low level of father’s education, feeling of loneliness and depressive symptoms, were found to contribute significantly to the prevalence of smoking among these street children. Studies in India and Philippines documented that street children, who are usually out-of-school youths, are more vulnerable to risky practices, such as increased smoking and other forms of substance abuse especially in the face of weak family bonds and harsh survival realities [39,40]. Adequate social support and efforts to integrate youths with their families may serve as one strategy to reduce tobacco smoking [39]. Odukoya et al. (2016) reported the highest prevalence of tobacco smoking in this review, focusing on out-of-school youths in motor parks around Lagos, South Western Nigeria [27]. Consistent among the general population and gender-specific groups, this high prevalence highlights this vulnerable population of out-of-school youths in this geopolitical zone. Out-of-School youths are usually least prioritized during tobacco smoking research and control programs [23,27] and may lack opportunities to benefit from formal health education and antismoking campaigns, when compared to in-school youths [41].

A noteworthy finding was that the lowest prevalence values of tobacco smoking in this review was 0.2%, in a rural setting across Osun State, South West, Nigeria [42], and the highest Prevalence was 32.5% in an urban population in Lagos, South West, Nigeria [27]. However, generally throughout the review, there were no clear-cut demarcations in the prevalence of tobacco smoking within rural or urban communities apart from the one depicted by the range of prevalence. This finding, however, follows the systematic review conducted across Sub-Saharan African countries [35], showing no consistent disparity in smoking prevalence between rural and urban populations. For example, certain studies in rural settings reported high prevalence of tobacco smoking among adolescents [23,31,43] and higher prevalence in rural areas of the United States and India [44–46]. However, other studies in Poland and Peru showed that the prevalence of tobacco smoking among adolescents was higher among the urban population [47,48]. Hence, tobacco control policies should be strengthened across all Nigerian societies regardless of geography or existing health or socioeconomic inequalities.

Peer influence was the most common risk factor described among the studies reviewed [20,23,27,43,49–54]. This is consistent with one study among South African adolescents that showed that peer smoking had the strongest influence on smoking lifestyle [55]. Also, family conditions, such as low parental education, polygamy, not living with parents, having a parent who smokes and having divorced or separated parents, were additional risk factors [24,25,27,43,49,52–54,56]. One study among Australian youths documented that overt pressure from older family members to initiate smoking was uncommon, but the majority were influenced by the availability of tobacco owned by older relatives in their homes, smoking practices and techniques of similarly aged family members [57].

Stress, loneliness and depressive symptoms were the most common psychosocial risk factors for tobacco smoking [20,31,58], consistent in one study that focused on tobacco smoking among adolescents in seven African countries [59]. This review identified that young people who abuse other substances, such as alcohol and marijuana, or who consume other forms of tobacco, Excluding smoking, were at risk of tobacco smoking [23,27,31,60]. Since psychosocial problems among young people are usually due to physical, emotional and sexual abuse and neglect, they usually resort to tobacco smoking or other forms of substance abuse as a coping mechanism to ameliorate their condition [61]. However, psychosocial problems among adolescents are real and the access and availability of friendly supportive systems are needed to limit the use of harmful coping behaviors like smoking. Since adolescents involved in cigarette smoking are usually involved in alcohol or other forms of substance abuse, a widespread multi-component control strategy may effectively reach this high-risk population [59,62].

Media advertisements and increasing age were identified as risk factors for tobacco smoking. [24,25,27,49,50,53,56]. Similar findings among rural South African adolescents revealed that Increasing age and seeing actors smoke on television had significant associations with smoking [63]. Television actors usually serve as role models for young people, and they may subtly motivate adolescents to consider smoking as a socially desirable activity [63]. One study among American adolescents revealed that the temptation to experiment with tobacco was increased among those exposed to pro-tobacco advertisements [64]. Another study among North African adolescents documented that 98% were exposed to at least one form of tobacco advertisement, which was associated with increased susceptibility to smoking initiation and current smoking [37]. The health hazards of smoking and the impact on quality of life should be the primary focus on tobacco control initiatives for in-school and out-of-school youths.

This study has limitations. First, due to the heterogeneity of methodology and data, an analysis of the trends of tobacco smoking among youths could not be implemented. Indeed, future studies, which consider the adoption of the Nigerian National Tobacco Control Act of 2015, can incorporate this control measure and assess its impact on tobacco prevalence.

Second, since no meta-analysis was performed in this study, no quantitative data analysis is available for further interpretation.

Third, although this review exhausted the scientific literature, including three databases, Google Scholar search, GYTS, government document review and direct communication with authors, we cannot overlook the fact that some publications may have been missed. However, this review is a valuable addition to existing literature and necessary for policy formulation and future monitoring of the impact of tobacco control measures. This also provides insight into the common challenges that African researchers encounter, leading to a paucity of health research productivity in Africa [65].

## Conclusions

This systematic review on the prevalence and associated determinants of tobacco smoking among Nigerian youths provides an adequate picture of this burden in the most populous country in Africa. Hence, all stakeholders should engage in concerted efforts to target both in-school and out-of-school youths in tobacco control strategies. Future research can explore the implementation of existing tobacco laws and the associated effect on trends of tobacco prevalence in Nigeria.

## Supporting information

S1 Table (DOCX)
